# Recalibrated FRIEND equation for peak oxygen pulse is accurate in endurance athletes: the NOODLE study

**DOI:** 10.1038/s41598-024-73730-z

**Published:** 2024-10-04

**Authors:** Przemysław Kasiak, Tomasz Kowalski, Andrzej Klusiewicz, Ryszard Zdanowicz, Maria Ładyga, Szczepan Wiecha, Artur Mamcarz, Daniel Śliż

**Affiliations:** 1 3rd Department of Internal Medicine and Cardiology, Medical University of Warsaw, Warsaw, Poland; 2 Doctoral School, Medical University of Warsaw, Warsaw, Poland; 3 Department of Physiology, Institute of Sport - National Research Institute, Warsaw, Poland; 4 Faculty of Physical Education and Health, Jozef Pilsudski University of Physical Education in Warsaw, Branch in Biala Podlaska, Biała Podlaska, Poland; 5 Clinical Cardiology Department, National Medical Institute of the Ministry of Interior and Administration, Warsaw, Poland

**Keywords:** Peak oxygen pulse, Endurance athletes, Cardiopulmonary exercise test, Cardiorespiratory fitness, Reference values, O_2_pulse, Cardiology, Health care, Medical research, Physiology, Cardiovascular biology, Circulation, Respiration

## Abstract

Peak oxygen pulse (O_2_P_peak_) is an important index of cardiorespiratory fitness (CRF). The FRIEND database is a global source of reference values for CRF. However, no reference equation is tailored for endurance athletes (EA) to predict O_2_P_peak_. Here, we adjusted the well-established FRIEND equation for O_2_P_peak_ to the characteristics of the EA population. 32 (34.0%) female EA and 62 (66.0%) male well-trained EA underwent maximal cardiopulmonary exercise test on a treadmill. V̇O_2max_ was 4.5 ± 0.5 L min^−1^ in males and 3.1 ± 0.4 L min^−1^ in females. O_2_P_peak_ was 23.6 ± 2.8 mL beat^−1^ and 16.4 ± 2.0 mL beat^−1^ for males and females, respectively. Firstly, we externally validated the original FRIEND equation. Secondly, using multiple linear regression, we adjusted the FRIEND equation for O_2_P_peak_ to the population of EA. The original FRIEND equation underestimated O_2_P_peak_ for 2.9 ± 2.9 mL beat^−1^ (*P* < .001) in males and 2.2 ± 2.1 mL beat^−1^ (*P* < .001) in females. The updated equation was 1.36 + 1.07 (23.2 · 0.09 · age − 6.6 [*if female*]). The new equation explained 62% of the variance and significantly predicted O_2_P_peak_ (R^2^ = 0.62, β = 0.78, *P* < .001). The error of the EA-adjusted model was 0.1 ± 2.9 mL beat^−1^ (*P* = .82) and 0.2 ± 2.1 mL beat^−1^ (*P* = .65) for males and females respectively. Recalibration of the original FRIEND equation significantly enhances its accuracy among EA. The error of the EA-adjusted model was negligible. A new recalibrated equation should be used to predict O_2_P_peak_ in the population of EA.

## Introduction

Maximal oxygen uptake (V̇O_2max_) obtained from a cardiopulmonary exercise test (CPET) is the gold-standard measure of cardiorespiratory fitness^1^. However, other variables also provide valuable information and give a more precise and comprehensive view^2^. One such index is the peak oxygen pulse (O_2_P_peak_), the ratio of V̇O_2max_ and maximal heart rate (HR_max_)^3,4^. The athletic population exhibits different physiological characteristics than patients or the untrained healthy population^5^. A key component of the O_2_P_peak_, i.e. V̇O_2max_ is significantly higher in endurance athletes^6^. Similarly, the HR_max_ declines slower with age in endurance athletes^7^.

Therefore, we should underline several sex and fitness-driven interactions in endurance athletes that could influence the O_2_P_peak_^5^. Athletes in dynamic endurance disciplines, such as running, could observe a more rapid increase in O_2_P_peak_ than untrained individuals^5^. Cardiorespiratory fitness, and particularly O_2_P_peak_, deteriorates slower with age or during periods of untraining^5,8^. Moreover, females adapt differently to males to endurance training and the changes include hormone levels, athletic performance, electrocardiogram, and cardiovascular imaging^9^. Other cardiac adaptations resulting from endurance training include increased cardiac output, improved cardiac dimensions, and better contractility^10,11^. All those changes lead to bigger stroke volume and higher oxygen transport^10^. O_2_P_peak_ mirrors the function of the athlete’s heart which works as a synergistic unit and O_2_P_peak_ is especially related to the stroke volume. The stroke volume is higher in endurance athletes^12^. Summing up, O_2_P_peak_ should also be higher in endurance athletes than in normal, untrained subjects^12–14^. All above changes in cardiac adaptations to exercise suggest that the variability of O_2_P_peak_ could be more complex than V̇O_2max_ or HR_max_ alone^4^. Therefore, understanding differences in cardiac physiology between athletes and untrained individuals is crucial to further investigate relationships between exercise variables properly.

Despite its several benefits, CPET is not always possible^15^. The most important limitations that should be acknowledged are the lack of qualified personnel, the high cost of procedures, and no properly equipped diagnostic centers^15,16^. Therefore, numerous prediction equations are developed to estimate cardiorespiratory fitness indirectly^17^. The comparison of values recorded during CPET to predicted is used to stratify the individual’s fitness level^18^. However, there is limited knowledge about predictions of O_2_P_peak_ in endurance athletes.

The “Fitness Registry and the Importance of Exercise: A National Data Base” (FRIEND) provides universal reference values for cardiorespiratory fitness^6^. So far, there have been only a few studies that tried to develop prediction equations for O_2_P_peak_^4,19^. However, in the majority, they have limited practical application besides the derivation group. Those equations include variables that most laboratories do not measure in standard CPET, e.g. systolic blood pressure or resting heart rate^19^. Furthermore, previous equations were usually developed from populations of a few hundred people. Arena et al. proposed the prediction equation for O_2_P_peak_ from the FRIEND database for the general population based on widely available variables^4^. There are no more prediction equations for O_2_P_peak_ which include universal, easy-accessible demographic variables. Moreover, there is a lack of prediction equations for O_2_P_peak_ for endurance athletes despite that O_2_P_peak_ is a useful index in trained individuals^12^.

Moreover, elite endurance athletes are subjected to constantly growing training loads and competition demands^20^. It leads to significant physiological adaptations and sometimes may increase the risk of adverse cardiovascular events^20,21^. Hence, a precise assessment of cardiorespiratory fitness specific to well-trained endurance athletes is of vital importance^22^. To properly apply prediction equations in sports diagnostics and for clinical purposes in endurance athletes, their accuracy must be first validated and adjusted^23^. Such validation and assessment of differences between observed and predicted data allows for a correct understanding of CPET results^24^.

This highlights the need to revise and adapt the standard reference equation for O_2_P_peak_ to the endurance athlete population. To address those issues, here we: (1) externally validated the well-established FRIEND prediction equation for O_2_P_peak_ on the cohort of endurance athletes and (2) recalibrated the prediction equation for O_2_P_peak_ for endurance athletes.

## Materials and methods

### Study design

We obtained approval from the Institutional Review Board of the Medical University of Warsaw (Pawińskiego 3C Street, 02-106 Warsaw, approval no. AKBE/277) for this study. Then, after written informed consent, we analyzed the data of well-trained endurance athletes admitted in 2022–2023 to the Institute of Sport—National Research Institute, Warsaw, Poland. We followed the Declaration of Helsinki and the STROBE statement for cross-sectional studies^25^.

Exercise tests were part of routine periodic performance evaluations during the season. The participants were elite athletes from national or development teams in endurance sports (medium or long-distance running, triathlon, ski mountaineering, cross-country skiing, and biathlon). All endurance athletes were in the 75th, 90th, or 95th percentile according to *Reference Standards for Cardiorespiratory Fitness* by Kaminsky et al.^6^

Only healthy adult individuals ≥ 18 years old underwent CPET. We ensured the health and safety of all endurance athletes by rigorous medical evaluation during their admission. Briefly, the health-related exclusion criteria were the same as in previous research from our lab^26^. The medical doctor assessed their 12-lead ECG, echocardiography, complete blood count, past medical history, and family history. The physician also performed auscultation, and neurological and orthopedic examinations to confirm the lack of any contraindication for maximal exercise tests. Moreover, all the endurance athletes had to deny smoking.

### Exercise tests

**Fig. 1:**
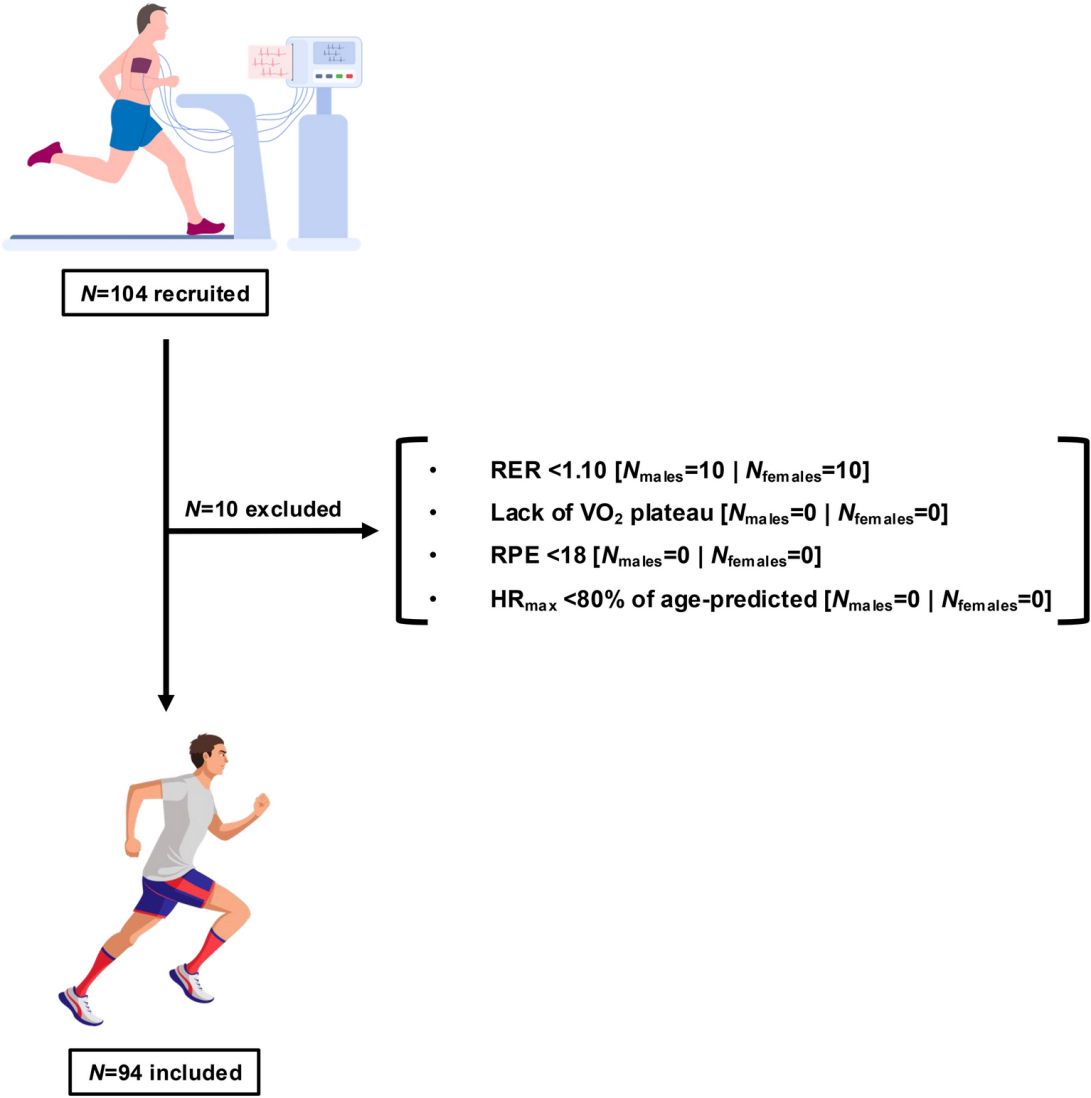
Flowchart for selection of participants. *Abbreviations*: RER, respiratory exchange ratio; VO_2_, oxygen uptake; RPE, rating of perceived exertion; HR_max_, maximal heart rate.

Participants performed maximal interval CPET in a step protocol on the treadmill (h/p/cosmos Saturn treadmill, h/p/cosmos sports & medical gmbh, Nussdorf-Traunstein, Germany). To ensure similarity with the derivation study we selected only running CPET. Endurance athletes were familiar with the specificity of running CPET, as everyone has performed it multiple times before. The test started with a brief warm-up of easy walking or jogging. The proper part began from the 8 km h^−1^ (for females) and 10 km h^−1^ (for males) and consisted of 4-min intervals. We increased the speed by 1.5–2.0 km h^−1^ for each interval. Running speed was individually adjusted for each CPET after consultation of an individual athlete with the supervising physiologist. We implemented a 60-s recovery between intervals. We set the treadmill inclination of 1.5% for the whole CPET protocol. Endurance athletes continued the grading interval protocol until CPET termination. All the following endpoints had to be fulfilled to consider the maximal effort: [1] 30-s plateau in V̇O_2_, [2] respiratory exchange ratio ≥ 1.10, [3] rating of perceived exertion ≥ 18, and [4] ≥ 80% of age-predicted maximal heart rate according to Fox equation (220-age [*in years*])^27^. Figure 1 presents the process of selecting participants who underwent maximal effort during CPET.

### Measured cardiorespiratory indices

We considered V̇O_2max_ as the highest 30-s stable oxygen uptake directly before the end of CPET. We measured all the gas exchange variables in the breath-by-breath method with the usage of Hans Rudolph V2 Mask (Hans Rudolph, Inc., Shawnee, KS, United States) and the Cortex MetaLyzer B3 (CORTEX Biophysik GmbH, Leipzig, Germany). We calibrated all the equipment for gasometry according to the manufacturer’s instructions. The participants wear Polar H10 (Polar Electro Oy, Kempele, Finland) chest straps to measure their heart rate (signal quality of 99.4%)^28^. We considered the HR_max_ as the highest value averaged in the 30-s intervals. Finally, we estimated the O_2_P_peak_ by dividing the V̇O_2max_ by HR_max_ and presented it as a mL beat^−1^^4^.

### Statistical analysis

We began the analysis by testing the data assumptions. For this purpose, we implemented the Shapiro–Wilk test and Q–Q plots. As all the data showed the parametric distribution, we presented continuous variables as mean ± standard deviation. We present categorical variables as numbers (percentages). In case of any missing variable, we exclude the participant from the analysis to ensure maximal data precision. The results presentation follows the 11th AMA *Manual of Style* guidelines^29^.

O_2_P_peak_ was the outcome variable, and age was an independent variable. The outcome and independent variable were the same as in the original FRIEND equation to ensure similarity between our study and the original derivation study. There were no interaction factors in the models, both basic and recalibrated. Further, we externally validated the FRIEND equation for O_2_P_peak_. We compared observed and estimated values using the Student t-test for independent means. Finally, we regressed the observed against the predicted peak O_2_P_peak_ and estimated the equivalent of the previous model. Adjusted coefficient of determination (R^2^) and root-mean-square error (RMSE) present regression results. On Bland & Altman plots, we visualized the performance of original and adjusted models.

We performed the post-hoc power analysis of the sample size in the G*Power Software (V3.1) for all applied statistical tests^30^. The whole population, subgroups of male endurance athletes or female endurance athletes reached power ≥ 0.8 and large effect size (d ≥ 0.8) for the Student t-test and linear regression^31^.

For calculations, we used SPSS Statistics (V29; IBM, Chicago, IL, USA) and we implemented GraphPad Prism (V10.2.2; GraphPad Software, San Diego, CA, USA) to derive figures. We considered two-tailed *P* < 0.05 as significant.

## Results

### Characteristics of participants

From 104 endurance athletes recruited for this study, 94 (90.4%) of them fulfilled all inclusion criteria. There were 32 females and 62 males. All the participants had > 4 years of competitive training experience. Briefly, O_2_P_peak_ for male endurance athletes equals 23.6 ± 2.8 mL beat^−1^, and for female endurance athletes, it was 16.4 ± 2.0 mL beat^−1^. Males achieved V̇O_2max_ of 4.5 ± 0.5 L min^−1^ and females achieved 3.1 ± 0.4 L min^−1^. In the majority, participants were in the 90th (N = 14, 14.9%) or 95th (N = 43, 45.7%) percentile for cardiorespiratory fitness. We presented the full characteristics of endurance athletes in Table 1.

**Table 1:** Description of the study population.

Variable	Total [N = 94]	Males [N = 62, 66.0%]	Females [N = 32, 34.0%]
Age [years]	27.5 ± 5.3	28.1 ± 5.5	26.4 ± 5.0
Weight [kg]	70.8 ± 12.1	76.1 ± 10.2	60.6 ± 8.3
Height [cm]	177.2 ± 9.3	181.2 ± 7.3	169.5 ± 7.9
BMI [kg m^−2^]	22.4 ± 2.5	23.2 ± 2.5	21.0 ± 1.8
HR_max_ [beats min^−1^]	189.6 ± 9.4	189.3 ± 9.7	190.1 ± 8.9
V̇O_2max_ [L min^−1^]	4.0 ± 0.8	4.5 ± 0.5	3.1 ± 0.4
V̇O_2max_ [mL kg min^−1^]	56.7 ± 8.6	59.2 ± 0.6	51.9 ± 6.3
V̇O_2max_ [percentile]			
95th	43 [45.7%]	25 [40.3%]	18 [56.3%]
90th	14 [14.9%]	10 [16.1%]	4 [12,5%]
75th	37 [39.4%]	27 [43.6%]	10 [31.2%]
RER	1.20 ± 0.06	1.20 ± 0.06	1.20 ± 0.06
f_R_ [breaths min^−1^]	62.0 ± 10.0	63.1 ± 9.8	59.8 ± 10.3
O_2_P_peak_ [mL beat^−1^]	21.1 ± 4.2	23.6 ± 2.8	16.4 ± 2.0
Predicted O_2_P_peak_ [mL beat^−1^]	18.5 ± 3.1	20.7 ± 0.5	14.2 ± 0.4
Recalibrated predicted O_2_P_peak_ [mL beat^−1^]	21.1 ± 3.3	23.5 ± 0.5	16.6 ± 0.5
Maximal speed [km h^−1^]	16.7 ± 2.1	17.3 ± 1.9	15.5 ± 1.9
Duration of test [minutes]	26.3 ± 3.8	26.9 ± 3.8	25.1 ± 3.7

We calculated O_2_P_peak_ by dividing the V̇O_2max_ by HR_max_. We estimated the predicted O_2_P_peak_ from the FRIEND equation and further recalibrated the original models to be adjusted for endurance athletes. V̇O_2max_ percentiles were calculated based on *Reference Standards for Cardiorespiratory Fitness* by Kaminsky et al.^6^.

*Abbreviations*: BMI, body mass index; HR_max_, maximal heart rate; V̇O_2max_, maximal oxygen uptake; RER, respiratory exchange ratio; f_R_, breathing frequency; O_2_P_peak_, peak oxygen pulse.

### Validation of original FRIEND equation

Briefly, the regression both for original and adjusted models explained 62% of the variance in the O_2_P_peak_ (R^2^ = 0.62, F(1, 92) = 147.1, *P* < 0.001). The RMSE was low and equals 2.6 mL beat^−1^. We found that previous and recalibrated models significantly predicted the O_2_P_peak_ to the same degree (β = 0.78). The original FRIEND equation underestimated O_2_P_peak_ in male endurance athletes for 2.9 ± 2.9 mL beat^−1^ (11.0%) and in female endurance athletes for 2.2 ± 2.1 mL beat^−1^ (12.3%). In direct comparison among male endurance athletes, predicted O_2_P_peak_ differed significantly for the original equation (t(122) =  − 8.06, *P* < 0.001). Moreover, the O_2_P_peak_ estimated from the original equation differed significantly from directly measured values also in females (t(62) = − 6.06, *P* < 0.001).

### Performance of the modified model

The difference between directly observed and estimated O_2_P_peak_ for the recalibrated model was negligible both in males (0.1 ± 2.9 mL beat^−1^, 1.1%) and in females (0.2 ± 2.1 mL beat^−1^, 2.4%). Furthermore, the recalibrated FRIEND equation outperformed the original model. In males, an updated equation did not present a significant difference between observed and estimated values (t(122) =  − 0.23, *P* = 0.82). As expected, a similar relationship occurred among female endurance athletes and the difference for the recalibrated model was insignificant (t(62) = 0.46, *P* = 0.65).

**Fig. 2:**
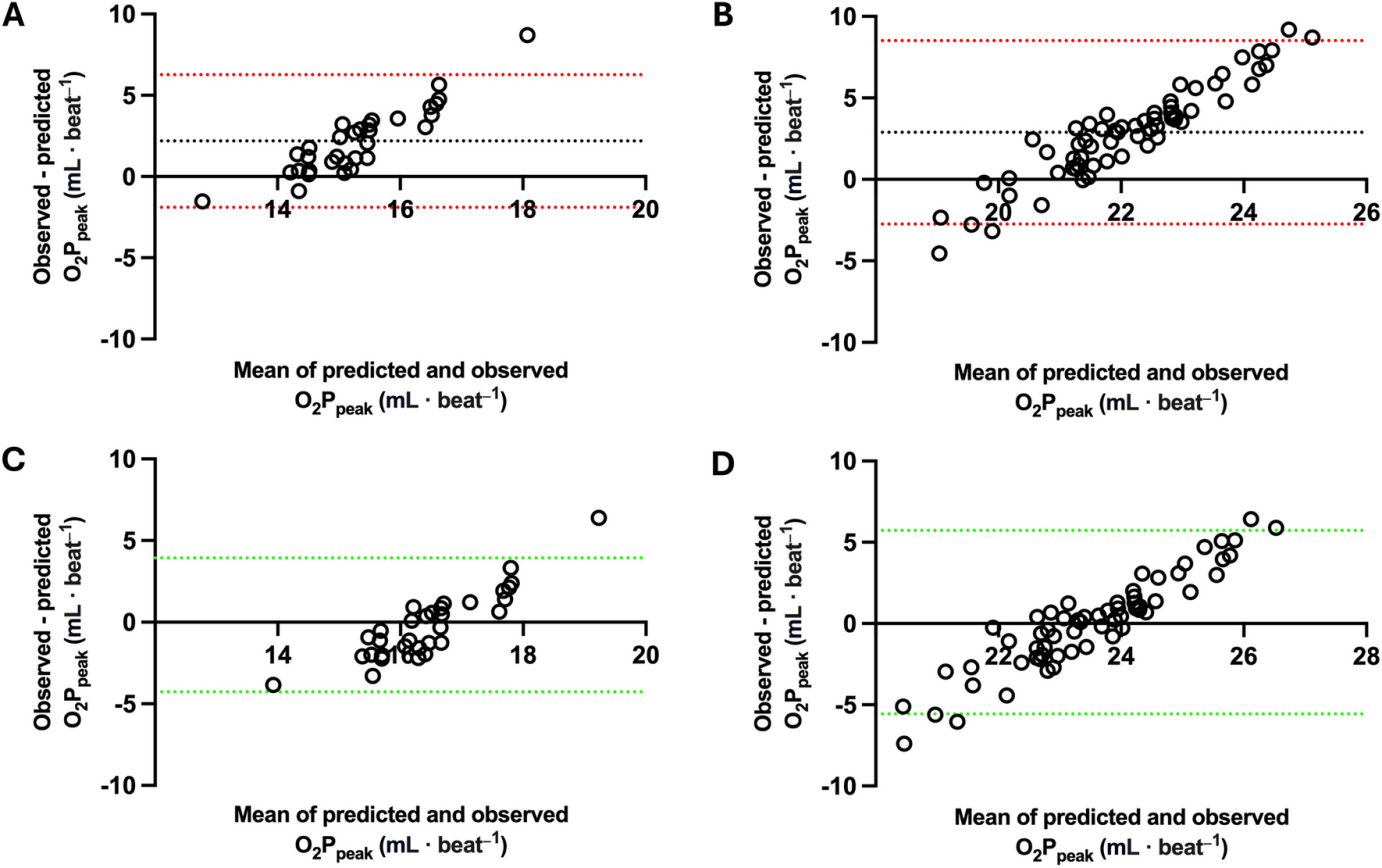
Comparison of observed and predicted O_2_P_peak_ for original and recalibrated FRIEND equations. *Abbreviations*: O_2_P_peak_, peak oxygen pulse. *Note*: In the Bland & Altman plots we compared observed and predicted O_2_P_peak_ for both equations. Panel A presents the accuracy of the original model for females and panel B presents the original model for males. Panel C presents the accuracy of the recalibrated model for females and panel D presents the accuracy of recalibrated model for males. Green and red dotted lines present upper and lower limits of agreement. The black dotted line on panels A and B presents the bias of the original equation. The bias line covers the X axis on panels C and D for the recalibrated equation because the error was minimal.

We present the original and recalibrated FRIEND equations for O_2_P_peak_ in endurance athletes in Table 2. Finally, we compared observed and predicted values for both equations on the Bland & Altman plots in Fig. 2.

**Table 2:** Recalibrated FRIEND equation for O_2_P_peak_ in endurance athletes.

Variable		Estimate	Standard error	β	95% CI		*P*-value
					UL	LL	
Intercept		1.36	1.65	–	− 1.92	4.64	.41
Original FRIEND equation	23.2–0.09 age [*in years*]–6.6 [*if female*]	1.07	0.09	0.78	0.90	1.25	< .001

*Abbreviations*: CI, confidence interval; UL, upper limit; LL, lower limit. The updated model should be inputted as follows: O_2_P_peak_ = 1.36 + 1.07 (23.2–0.09 age [in years]–6.6 [if female]). The model explained 62% of the variance in O_2_P_peak_ in endurance athletes (R^2^ = 0.62, F(1, 92) = 147.1, *P* < .001).

## Discussion

This study provides several important findings. Firstly, the general reference equation provides limited accuracy and consistently underpredicts O_2_P_peak_ in endurance athletes. Secondly, the recalibration with the adjustment of covariates significantly enhances the accuracy of predictions. Thirdly, we suggest a framework for adjusting the general reference equations for a population of endurance athletes. We highlight that there is not always a need to derive a completely novel equation for endurance athletes. The comparison of observed and predicted values with the usage of equivalents of the existing prediction models could be a valuable alternative to direct CPET for endurance athletes.

The FRIEND database includes several thousand individuals (the exact sample size depends on the study)^32,33^. Reference values from the FRIEND database constitute a universal reference point transferable across the laboratories and through the lifespan^34^. However, the FRIEND database in the majority included people with a moderate fitness level^35^. Thus, it may not be ideally suited for endurance athletes. Therefore, the FRIEND database is a valuable basis and starting point for predicting the cardiorespiratory fitness indices in endurance athletes. However, it requires some adjustments to the physiology of individuals with above-average fitness levels.

Previous studies indicate that the most appropriate way to estimate cardiorespiratory fitness in specific populations is to derive novel equations for them^17^. In the present study, we present a simpler approach. We do not derive a completely new model but only adjust the coefficient of the existing one. Bland & Altman plots (see Fig. 2) support the idea of such a recalibration. As expected, the original FRIEND equation significantly underestimated the O_2_P_peak_ in the cohort of well-trained endurance athletes (see panels A and B of Fig. 2). It was developed based on the untrained subjects and endurance athletes exhibited higher O_2_P_peak_^4^. However, panels C and D with adjusted models show minimal bias. The statistical analysis results confirmed the relationship visible in Fig. 2.

The observed and predicted values were significantly different for the original FRIEND equation but not for the adjusted one. It should be emphasized, that the only change was adding a multiplier, without modifying the existing FRIEND equation. This innovative way of recalibration is the new method to indirectly predict cardiorespiratory fitness in endurance athletes, as it enables the correct interpretation of cardiorespiratory fitness in this population. The constant growth of the amateur endurance athletes population and training loads among elite endurance athletes underline the significance of our findings and the applicability of the presented approach^20^.

### Clinical implications

Oxygen pulse is a valuable component of CPET. Its role is invaluable in diseased populations as well as in physically fit individuals^36^. Cardiovascular diseases do not omit athletes who are exposed to significant training and competition overloads^21^. The results of the present study facilitate confirmation of whether an endurance athlete should not be suspected of any pathology. Comparison of the achieved CPET results with the predictions from the adjusted FRIEND model verifies athletic cardiorespiratory fitness^17^. Moreover, retrospective evaluation of past CPET results considering the estimated values adds significant value to the clinician’s toolbox when assessing the heart of endurance athletes. We assume that significantly lower actual O_2_P_peak_ may suggest pathology whereas similar O_2_P_peak_ suggests normal cardiac function in endurance athletes, but these relationships should be investigated by future studies.

### Limitations

We must highlight some caveats of the present study to ensure its proper understanding. Even though the analyzed group fulfilled the required sample size criteria, it was strongly homogeneous. Our subjects were highly trained endurance athletes, therefore populations of different training statuses, i.e. novice amateur endurance athletes, may require further adjustment of the O_2_P_peak_ model. Moreover, our participants were White Europeans and ethnicity could contribute to cardiorespiratory fitness^37^. As this is the first study to present such an approach to recalibrating a well-established prediction equation, we recommend further confirmatory studies on more demographically diverse (especially regarding race and age) populations or groups of lower fitness status.

## Conclusions

To conclude, a recalibrated model for O_2_P_peak_ was highly accurate in well-trained endurance athletes. There was no need to change the original FRIEND equation. It was enough to calculate the equivalent of the FRIEND equation to enhance its precision among endurance athletes significantly. We recommend using the model to compare direct and estimated O_2_P_peak_ and to establish new reference values for O_2_P_peak_ in well-trained endurance athletes.

## Data Availability

The datasets generated during and/or analyzed during the current study are available from the corresponding author on reasonable request (Przemysław Kasiak; przemyslaw.kasiak@wum.edu.pl).
